# A131 ENDOSCOPIC SUBMUCOSAL DISSECTION OF COLORECTAL ADENOMAS AND EARLY ADENOCARCINOMAS: OUTCOMES FROM BRITISH COLUMBIA

**DOI:** 10.1093/jcag/gwac036.131

**Published:** 2023-03-07

**Authors:** B Zhao, H J Kim, R Trasolini, D Chahal, E Lam

**Affiliations:** 1 Medicine; 2 Gastroenterology, University of British Columbia, Vancouver, Canada; 3 Gastroenterology, Harvard University, Cambridge, United States

## Abstract

**Background:**

Endoscopic resection is the standard of care for the management of colorectal polyps. Larger and more complex polyps require endoscopic mucosal resection (EMR). While complications have been low, EMR is often piecemeal, resulting in indeterminant margins and often a higher recurrence rate. Endoscopic submucosal dissection (ESD) is an advanced endoscopic resection technique with a higher rate of en bloc resection. While more data exist for the resection of gastric lesions with ESD, ESD is becoming more widely used in western countries for the resection of colorectal lesions.

**Purpose:**

The purpose of this study is to report on the outcomes and rates of complications for colorectal ESD completed in a tertiary centre in British Columbia.

**Method:**

All colorectal ESD was completed by a senior therapeutic endoscopist who has previously received training in Japan. Retrospective data were collected on all colorectal ESD procedures done in St. Paul’s Hospital from July 11th, 2016, when the procedure first became available, to Aug 30th, 2022. Inclusion criteria were all adults who have undergone ESD for resection of a colorectal lesion. Exclusion criteria were patients younger than 18. Data collected included demographic variables, polyp characteristics, procedural outcomes, and complications.

**Result(s):**

A total of 39 ESD procedures were completed. The mean size of the resected lesion was 30.4 mm (range: 5 – 60 mm). Technical success, defined as successful resection of all polypoid tissue, was achieved in 35/39 procedures (89.7%). En-bloc resection was achieved in 27/35 (77.1%) of the completed ESD. The rate of R0 resection was 22/35 (62.9%). Curative resection, defined as technically successful ESD with R0 margin and no lymphovascular invasion, was achieved in 23/39 (59.0%) of the cases and the majority of the patients with non-curative resection that underwent endoscopic surveillance had no recurrence on follow-up. In our cohort, 3/39 (7.7%) patients had adenocarcinoma. None of the ESD resulted in any intra-procedural or delayed perforation. 3/39 (7.7%) patients had clinically significant post-endoscopic resection bleeding. Out of 24 patients that completed follow-up, 4 (16.7%) had recurrence at the resection site that was managed endoscopically. 4/39 (10.3%) of patients required surgery post-ESD.

**Conclusion(s):**

In our cohort, ESD is an effective endoscopic resection modality for the management of colorectal adenomas and early adenocarcinoma with a high rate of technical success and low rates of complications. Although the rate of curative resection was low, most were the result of R1 or Rx resection and a majority of the follow-ups in this subgroup demonstrated no further recurrence. The rate of en bloc resection is high, especially given the average size of adenomas in this cohort. Although ESD requires high technical proficiency, its favorable outcomes and low complication rates make ESD highly feasible for the resection of colorectal lesions.

**Please acknowledge all funding agencies by checking the applicable boxes below:**

None

**Disclosure of Interest:**

None Declared

